# Books and Magazines

**Published:** 1908-04

**Authors:** 


### Books and Magazines.

SURGICAL THERAPEUTICS.—By Emory Lanphear, M. D., Ph. D., LL. D., St. Louis, Mo., Professor of Surgery, Hippocratean College of Medicine, St. Louis, etc., Chicago. Clinic Publishing Co., 1907. Cloth, 375 pages. Price not stated.

Dr. Lanphear says that many books tell how to operate, but not one describes the proper management of the patient *without* operation,—a kind of bloodless surgery, I suppose. His aim is, therefore, to give the "non-operative treatment of surgical conditions," and to describe some important points omitted from the more elaborate works on surgery. The contents are arranged alphabetically for convenient reference.

Nervous and Mental Diseases.—By G. P. Head, M. D., and H. T. Patrick, M. D. This is Vol. X of the Year Book Co.'s Practical Medicine Series for 1907, and completes the set. Price, $1.25. The series of ten volumes, $10.

The antecedent volumes having been reviewed in some detail this one requires no further mention.

---

Practical Fever Nursing.—By Edward C. Register, M. D., Professor of the Practice of Medicine in the North Carolina Medical College. Octavo volume of 352 pages, illustrated. Philadelphia and London: W. B. Saunders Company, 1907. Cloth, $2.50 net.

Dr. Register's book is the outcome of his observation and experience. He has felt the lack of sufficient knowledge on the part of "trained nurses," since they have become an indispensable adjunct to the doctor in the practice of medicine. She must be trained, and also educated. In fact, she must be a pretty good "doctor" herself, since so much is left to her knowledge and discretion in the absence of the doctor. She must know something except dietetics, how to prepare "nourishment," and how to take temperature, etc. The book is written especially for the nurse, although many doctors of my acquaintance would do well to read it, for it tells of many things that some of them are not up on. In fact, the nurse must know pathology and have some acquaintance with the general principles of therapeutics. I commend the work as well worth while.

---

Cosmetic Surgery—The Correction of Featural Imperfections.—By Charles C. Miller, M. D. Including the description of a variety of operations for improving the appearance of the face. 136 pages. 73 illustrations. Prepaid, $1.50. Published by the author, 70 State St., Chicago, Ill.

Dr. Miller declares that "there is today a well-established demand for skillful featural surgeons," and feels that he can do the medical profession no greater service than to offer them the results of his experience. That is the purpose of this neat little book. It tells one how to make big outstanding ears lie flat; how to reconstruct or repair an ear that has been lost or mutilated; how to correct folds, bags and wrinkles of the skin about the eyes; how to reduce a hump nose; how to take the conceit out of a tilted nose; how to make subcutaneous injections of paraffin for various purposes—wrinkles, particularly; how to correct the everted and the inverted lip; how to make a big mouth smaller and a small mouth larger; how to make dimples; how to do numerous things to make a person better looking. It is illustrated by numerous cuts showing the deformities and each step in the operation for its correction. It is well worth the price asked for it.

